# Augmentative and Alternative Communication as an Ecological Window on Neglect-Related Spatial Asymmetry After Hemorrhagic Stroke: A Longitudinal Case Report

**DOI:** 10.3390/brainsci16050456

**Published:** 2026-04-24

**Authors:** Carmela Rifici, Rosaria De Luca, Francesco Corallo, Sabrina Miceli, Santina Caliri, Andrea Calderone, Rosalia Calapai, Alessio Mirabile, Maria Pagano, Angelo Quartarone, Rocco Salvatore Calabrò

**Affiliations:** IRCCS Centro Neurolesi Bonino Pulejo, 98124 Messina, Italy; carmela.rifici@irccsme.it (C.R.); rosaria.deluca@irccsme.it (R.D.L.); francesco.corallo@irccsme.it (F.C.); santina.caliri@irccsme.it (S.C.); rosalia.calapai@irccsme.it (R.C.); alessio.mirabile@irccsme.it (A.M.); angelo.quartarone@irccsme.it (A.Q.); roccos.calabro@irccsme.it (R.S.C.)

**Keywords:** hemorrhagic stroke, spatial neglect, augmentative and alternative communication, eye-tracking, ecological assessment, neurorehabilitation, communication disorders, P300, case report

## Abstract

**Highlights:**

**What are the main findings?**

- In this index case, global quantitative measures showed modest early improvement followed by stabilization, whereas serial eye-tracking data across 21 analyzable sessions showed consistently adequate guided calibration but reduced spontaneous visuospatial exploration.
- Task-based AAC performance was lower and more unstable in the more exploratory Stars task (mean 2.14 hits; 5/21 zero-hit sessions) than in Bow-Target (mean 3.48 hits; 1/21 zero-hit session), while free-exploration heatmaps frequently showed rightward clustering and reduced left-hemifield exploration.

**What are the implications of the main findings?**

- Eye-gaze AAC may function as a structured ecological observational context in severely impaired patients, particularly when formal neglect assessment is limited, but this single case does not validate AAC as a stand-alone diagnostic tool.
- Standardized AAC-derived spatial metrics and direct comparison with gold-standard neglect measures remain necessary before any formal monitoring or phenotyping role can be defined.

**Abstract:**

Background/Objectives: Spatial neglect after stroke may be difficult to characterize in patients with severe motor, cognitive, and communication impairment. Augmentative and alternative communication interfaces require visual scanning and intentional selection and may therefore provide an ecological context in which lateralized visuospatial behavior becomes clinically observable. Methods: A 58-year-old man with a unilateral right-hemisphere hemorrhagic stroke underwent serial assessment at baseline before training, at the end of 24 AAC sessions delivered over 2 months in addition to standard neurorehabilitation, and at 1-month follow-up. Measures included cognitive functioning, behavioral responsiveness, global disability, bedside communication status, and P300 latency. The AAC/eye-tracking intervention also generated process data across 21 analyzable sessions, including calibration quality, free-exploration heatmaps, and performance in the Stars and Bow-Target tasks. Results: Global measures showed modest early improvement followed by stabilization. Cognitive functioning improved from 2 to 3 and remained stable, behavioral responsiveness increased from 7 to 10 and then to 11, bedside communication increased from 7 to 9 and remained stable, and P300 latency decreased from 393 to 350 and then to 351 ms, whereas global disability remained unchanged at 25 throughout. Calibration was at least good in all quadrants and never scored 0. Performance was lower and more unstable in Stars than in Bow-Target. Heatmaps showed rightward clustering, reduced left-sided exploration, and limited whole-screen scanning. Conclusions: AAC/eye-tracking did not provide formal diagnostic proof of neglect, but it supported ecological recognition of a neglect-like lateralized exploratory pattern under less guided conditions.

## 1. Introduction

Spatial neglect is one of the most frequent cognitive sequelae of stroke and remains strongly associated with poorer functional recovery, reduced participation in daily activities, prolonged inpatient rehabilitation, and greater caregiver burden [1,2]. Contemporary longitudinal rehabilitation studies also indicate that neglect is not a transient epiphenomenon but a clinically meaningful predictor of worse global outcome, especially when it is not identified early or when its expression is underestimated during recovery [3]. This relevance extends beyond the classic image of a patient ignoring one side of extrapersonal space. In routine practice, neglect often disrupts mobility, self-care, reading, communication, and engagement with rehabilitation tasks, thereby influencing both safety and treatment responsiveness.

The syndrome is heterogeneous. It may involve personal, peripersonal, or extrapersonal space; different sensory and motor modalities; and distinct perceptual, representational, or motor-intentional components [4]. This heterogeneity has direct consequences for assessment. No single bedside task can adequately represent the full clinical spectrum, and the psychometric literature continues to show important variability in the sensitivity and construct coverage of available tools [5]. Current recommendations accordingly advocate structured screening pathways and multidisciplinary awareness, yet international survey data suggest that neglect is still inconsistently screened and incompletely characterized in everyday post-stroke care [6,7]. As a result, patients may show clinically relevant lateralized behavior in routine activity even when formal testing is sparse, delayed, or only partially informative.

This gap between impairment-based testing and real-world behavior has long motivated the development of functional and ecological approaches. Conventional paper-and-pencil tasks may be useful for documenting a spatial bias, but they do not necessarily capture how that bias affects dressing, wheelchair navigation, object use, interaction with caregivers, or goal-directed communication [8]. Functional instruments such as the Catherine Bergego Scale were designed precisely to bridge this divide by anchoring neglect to everyday activity and therapist observation [9]. Even so, observational under-documentation remains a problem in rehabilitation records, and recent work suggests that clinical assessment behavior is still variable across services and disciplines [10,11]. Mixed-methods evidence further reinforces the point that neglect is expressed at the level of activity and participation, not only on decontextualized tests [12].

For this reason, ecological and technology-assisted approaches have received growing attention. Virtual reality, immersive environments, augmented reality, and eye-tracking paradigms can embed visuospatial exploration within purposeful tasks and may therefore reveal spatial asymmetries that are less apparent in static formats [13,14]. Recent studies indicate that digital environments can capture the multidimensional nature of neglect-related behavior, including exploratory strategy, task dependence, and adaptation to changing spatial demands [15,16]. Free-exploration paradigms in augmented reality point in the same direction and support the value of structured yet naturalistic observation when clinicians seek to understand how patients actually deploy attention in space [17]. These approaches do not replace clinical examination, but they help clarify why the ecological context of a task can influence the visibility of a lateralized deficit.

Augmentative and alternative communication (AAC) is usually discussed as a support for communicative participation rather than as an assessment environment. However, this distinction may be less rigid in severe neurological conditions. AAC has been used for adults with acquired neurological disorders for many years [18]. In post-stroke aphasia and other acquired brain injuries, high-technology AAC can extend expressive options and support interaction when spoken language, writing, or motor output are reduced [19,20]. Contemporary cognitive-communication rehabilitation guidelines also place AAC within the management of severe brain injury, particularly when patients need structured tools to sustain purposeful exchange [21]. Observational and implementation studies have described its feasibility and acceptance in adults with traumatic or severe acquired brain injury [22,23]. A recent neurorehabilitation case series likewise suggested that AAC may have practical relevance even in highly complex clinical conditions [24].

From a cognitive standpoint, AAC interfaces require more than simple message display. They require visual search, attentional allocation, target discrimination, purposeful selection, and repeated interaction within an organized spatial array. Accordingly, AAC is not presented here as a validated neglect assessment paradigm. This exploratory longitudinal case report examined whether a digital eye-gaze AAC context, combined with serial eye-tracking process data, could provide clinically useful ecological information about lateralized visuospatial behavior in a patient with hemorrhagic stroke whose severe motor, cognitive, and communication limitations constrained conventional assessment.

## 2. Case Presentation and Methods

### 2.1. Case Presentation

This report was designed as an exploratory longitudinal clinical case study based on routinely collected rehabilitation data and corresponding narrative clinical documentation. The index case and the contextual cases were identified within the same institutional dataset and had been assessed at three repeated time points labeled T0, T1, and T2. All numerical values were checked against the institutional database and source documentation before tabulation. The report is descriptive rather than inferential and is framed around serial clinical observation and process-level AAC data rather than hypothesis testing.

The index case was a 58-year-old man with 13 years of education who underwent inpatient neurorehabilitation after a unilateral right-hemisphere hemorrhagic stroke involving the fronto-temporo-parietal region. Pharmacological treatment during the rehabilitation phase included Olmesartan 20 mg, Baclofen 25 mg, and Desvenlafaxine 50 mg. T0 corresponded to pre-training baseline. T1 corresponded to the end of a 2-month AAC treatment block consisting of 24 sessions delivered three times per week in addition to standard neurorehabilitation. T2 corresponded to one-month follow-up after completion of the AAC block.

The same dataset contained three additional patients assessed at the same three time points with the shared clinical and neurophysiological variables. These patients were not treated as controls and were not matched for lesion characteristics. Instead, they served only as a descriptive contextual benchmark. They underwent structured low-tech paper-based AAC assessment using communication grids rather than the digital eye-gaze communicator used in the index case. Their mean age was 56.3 ± 9.0 years; two were male and one female; education was 13 years in all three cases.

### 2.2. Clinical and Communication Measures

Clinical status over time was described with the Levels of Cognitive Functioning (LCF), the Coma Recovery Scale-Revised (CRS-R), and the Disability Rating Scale (DRS). CRS-R is a standardized behavioral instrument widely used to assess auditory, visual, motor, verbal, communication, and arousal functions in patients with severe brain injury [25]. Its diagnostic utility and the influence of examiner expertise have been documented in later methodological studies [26], and total score thresholds have been explored to improve sensitivity to conscious awareness in clinically complex populations [27]. In the present case, CRS-R was used as a serial descriptive measure of behavioral responsiveness rather than as a stand-alone diagnostic label.

DRS was included as the available global disability index. It was developed to track disability from coma to community outcome and remains useful when clinicians need a broad summary of functional dependence across recovery [28]. LCF, by contrast, offers a staged description of cognitive and behavioral recovery, and prior work has supported its reliability and validity for longitudinal monitoring in severe brain injury settings [29,30]. Taken together, these instruments provided complementary information on global disability, responsiveness, and staged cognitive-behavioral change. Because they are not interchangeable and because several of them are ordinal or staging-based, their trajectories were interpreted descriptively rather than collapsed into a single summary index.

The communication variable available in the dataset was recorded as the Global Communication Scale (GC), a clinician-rated bedside index of verbal and non-verbal communicative exchange. No retrievable scoring manual, psychometric validation study, or standardized computation procedure was available in the source record. Accordingly, GC was treated strictly as a pragmatic descriptive clinical indicator and not as a formal validated outcome measure. GC scores are reported only to describe the direction of change over time and are not used to support generalized psychometric or inferential claims.

### 2.3. Neurophysiological Measure

P300 latency, expressed in milliseconds, was the only neurophysiological variable available for longitudinal analysis. In clinical neurophysiology, the P300 event-related potential is commonly interpreted as reflecting aspects of stimulus evaluation and attentional processing, although its precise meaning depends on task structure and patient state [31]. Studies in stroke populations, including hemorrhagic cases, suggest that P300 latency may provide useful complementary information regarding higher-order information processing, but it does not by itself define the spatial organization of overt behavior [32]. Accordingly, P300 latency is treated here only as a non-specific marker of stimulus evaluation speed and higher-order cognitive processing efficiency. Because amplitude, electrode montage, topography, and task details were not available, the present data do not permit any inference about hemispheric dysfunction, spatial attention allocation, or neglect mechanisms.

### 2.4. AAC Device and Eye-Tracking Procedure

AAC/eye-tracking data were acquired with a Tobii Dynavox eye-gaze communication system during the AAC treatment block. Available digital process data were analyzable for 21 sessions. Each recorded session began with calibration, during which a visual target appeared first centrally and then in the upper-right, lower-right, upper-left, and lower-left quadrants. Calibration quality was coded as 0 = poor, 1 = good, and 2 = perfect.

After calibration, the patient underwent a phase of free visual exploration, generating a summary screen/heatmap of explored areas, followed by two structured tasks. In Stars, a large star appeared in a random location on a black background and had to be fixated until it disappeared and reappeared elsewhere. In Bow-Target, the patient fixated a target to hit it, with progressive increases in the number of targets across levels. A Blocks-based preliminary training phase was completed in all recorded sessions, supporting the practical feasibility of continued device use even in the setting of severe acquired brain injury.

These procedures were not used as a formal neglect battery. Rather, they provided serial process data on guided target engagement, free exploration, and task performance within a structured ecological AAC context. The three contextual cases did not undergo this digital procedure.

### 2.5. Descriptive Analysis

All analyses were descriptive. The shared clinical variables were summarized at T0, T1, and T2 for the index case and as mean ± standard deviation for the contextual benchmark. The session-level eye-tracking dataset was summarized across the 21 analyzable sessions using calibration frequencies, task performance descriptive statistics (mean, median, range, and number of zero-hit sessions), and simple initial-versus-final phase comparisons. No inferential statistics were performed because of the single-case design, the very small contextual sample, and the exploratory aim of the report. Additional operational definitions and the supplementary statistical framework used for recoverable AAC/eye-tracking summaries are detailed in Supplementary Methods S1.

Quantitative and qualitative eye-tracking evidence were interpreted together but were not collapsed into an artificial composite score. This conservative approach allowed calibration adequacy, task-level performance, and heatmap patterns to inform the clinical interpretation without overstating diagnostic precision.

During manuscript preparation, generative artificial intelligence was used for the graphical generation/rendering of figures on the basis of author-defined content and verified study data. The authors critically reviewed and edited all figure outputs and take full responsibility for their accuracy and presentation. Generative AI was not used for data analysis, statistical computation, interpretation of findings, or formulation of conclusions.

## 3. Results

### 3.1. Longitudinal Quantitative Findings

**Figure 1:**
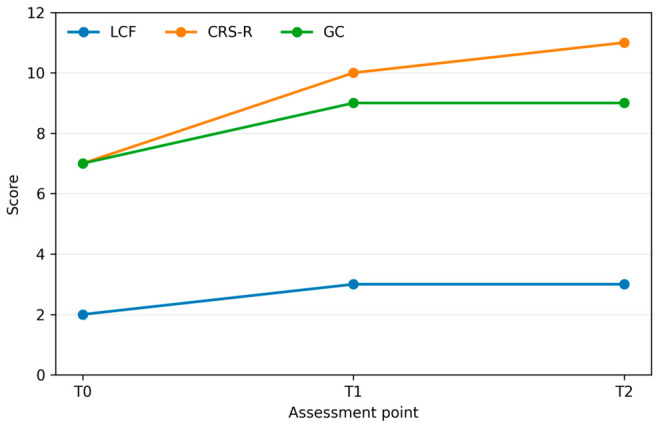
Serial Levels of Cognitive Functioning, Coma Recovery Scale-Revised, and Global Communication Scale values in the index case across T0, T1, and T2. Disability Rating Scale is not displayed because it remained stable at 25 across all assessments.

The longitudinal values for the index case are summarized in Table 1, and the corresponding trajectories of the shared clinical variables are shown in Figure 1. At baseline, LCF was 2, CRS-R was 7, DRS was 25, GC was 7, and P300 latency was 393 ms, consistent with severe global disability and delayed higher-order processing on the only available neurophysiological marker.

Between T0 and T1, the patient showed modest early improvement in selected domains: LCF changed from 2 to 3, CRS-R from 7 to 10, GC from 7 to 9, and P300 latency from 393 to 350 ms, whereas DRS remained 25. Between T1 and T2, the pattern was predominantly one of stabilization: LCF remained 3, GC remained 9, P300 latency remained essentially unchanged at 351 ms, and DRS remained 25, while CRS-R increased from 10 to 11. Taken together, the global quantitative profile is best described as modest early improvement followed by stabilization within persistently severe overall disability.

The recorded variables did not evolve in parallel. Behavioral responsiveness and staged cognitive functioning improved more than the global disability score, whereas the bedside communication index and P300 latency showed early change followed by plateauing. This non-parallel profile provided the context for interpreting the serial AAC/eye-tracking findings.

### 3.2. Descriptive Contextual Benchmark

**Figure 2:**
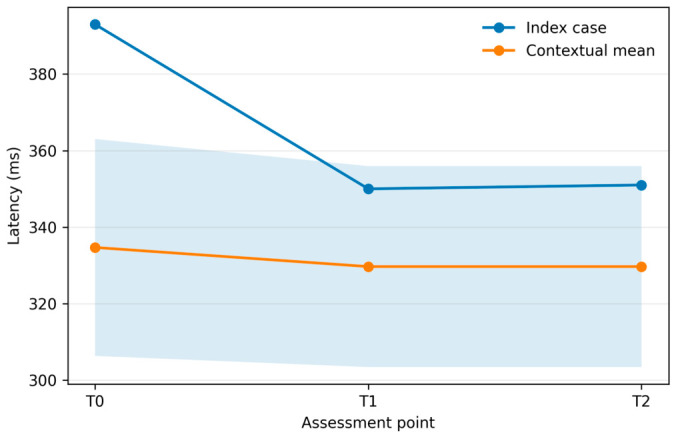
Serial P300 latency in the index case and in the descriptive contextual benchmark across T0, T1, and T2. The shaded band represents the contextual mean ± standard deviation and is shown for descriptive visualization only.

The three additional cases were retained only as a descriptive contextual benchmark and not as controls. The aggregated values of the descriptive contextual benchmark are shown in Table 2, and the corresponding P300 latency comparison between the index case and the contextual benchmark is displayed in Figure 2. In this very small benchmark, mean LCF increased from 1.67 ± 0.58 at T0 to 2.67 ± 0.58 at T1 and remained stable at T2. Mean CRS-R increased from 7.00 ± 3.46 at T0 to 9.67 ± 3.51 at T1 and remained unchanged at T2. Mean DRS remained 25.33 ± 1.15 at all time points. Mean GC increased from 8.00 ± 1.00 at T0 to 9.33 ± 1.53 at T1 and 9.67 ± 1.15 at T2. Mean P300 latency decreased slightly from 334.67 ± 28.36 ms at T0 to 329.67 ± 26.27 ms at T1 and remained unchanged at T2. Raw T0/T1/T2 values for the three contextual benchmark cases are reported in Supplementary Table S1.

The purpose of this benchmark was only to contextualize the broad scale of the index trajectory. No inferential comparison was attempted, and no language implying formal above-average or below-average performance is warranted in a sample of this size.

### 3.3. Serial AAC/Eye-Tracking Findings

Serial AAC/eye-tracking findings from the 21 analyzable sessions are summarized in Table 3. The Blocks preliminary training phase was completed in all sessions, supporting the clinical feasibility of repeated device use. Calibration was consistently at least good in every quadrant and never scored 0. The central calibration point was rated good in 19 sessions and perfect in 2. The upper-left and lower-left quadrants were rated good in all sessions. The upper-right quadrant was the best-performing sector, with 8 good and 13 perfect calibrations. The lower-right quadrant was rated good in 19 sessions and perfect in 2. Quadrant-level calibration frequencies, percentages, and exact binomial intervals for perfect calibration are reported in Supplementary Table S2.

**Figure 3:**
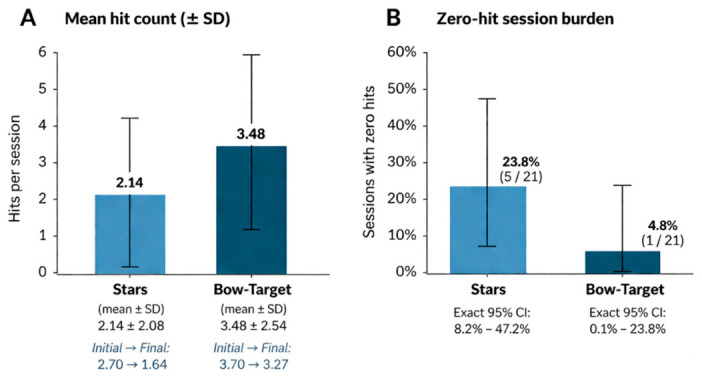
Descriptive statistical comparison of AAC task performance in Stars and Bow-Target across 21 analyzable sessions. Panel (**A**) shows mean hit count ± standard deviation for the two AAC tasks. Panel (**B**) shows the proportion of zero-hit sessions with exact 95% confidence intervals. Stars showed lower and more unstable performance than Bow-Target, consistent with greater vulnerability under exploratory task demands. Values are descriptive and should be interpreted within a single-case exploratory framework.

Task performance was more impaired and more variable under exploratory demands. In Stars, mean performance was 2.14 hits, median 1, range 0–8, with 5 of 21 sessions yielding zero hits. In Bow-Target, mean performance was 3.48 hits, median 2, range 0–9, with zero hits in 1 of 21 sessions. Initial-versus-final phase comparisons did not suggest sustained quantitative recovery in these AAC tasks: Stars decreased from 2.70 to 1.64 and Bow-Target from 3.70 to 3.27. Additional exact proportion intervals and variability descriptors derived from the recoverable task summaries are reported in Supplementary Table S3. A descriptive statistical comparison of task performance and zero-hit burden in Stars and Bow-Target is shown in Figure 3.

Qualitative review of the free-exploration heatmaps showed non-homogeneous spatial coverage, localized fixation clusters, frequent rightward lateralization, reduced exploration of the left hemifield, and limited evidence of systematic whole-screen scanning. Occasional leftward orienting occurred when stimuli were more salient, but the overall pattern remained fragmented and asymmetrical. Because some interface elements were not uniformly distributed across screens, the heatmap evidence was interpreted qualitatively and always integrated with the full clinical context. A representative phase-based qualitative coding of these heatmap patterns is provided in Supplementary Table S4, and representative early, intermediate, and final heatmaps are shown in Supplementary Figure S1.

Taken together, the serial eye-tracking data indicate relatively preserved guided orienting/calibration alongside reduced spontaneous visuospatial exploration/search efficiency. The pattern was therefore not most consistent with gross technical failure or a simple inability to fixate guided targets. Rather, difficulties emerged more clearly when the patient had to self-generate search, distribute attention across space, and maintain exploratory continuity. In this sense, the data are clinically compatible with a lateralized visuospatial attention disorder or neglect-like exploratory bias, especially under less guided conditions, while remaining insufficient on their own for a formal neglect diagnosis.

## 4. Discussion

This case is best interpreted as an exploratory proof-of-concept report rather than as evidence for a validated AAC-based neglect assessment strategy. The global quantitative profile showed modest early improvement followed by stabilization within persistently severe disability, whereas the serial eye-tracking dataset did not show corresponding normalization of visuospatial behavior. The key observation was that calibration for guided target engagement remained consistently adequate while spontaneous exploration and task performance remained inefficient and spatially unbalanced.

This distinction matters clinically. Consistently adequate calibration across all quadrants argues against explaining the later asymmetry by gross technical failure, an inability to engage guided targets, or persistently unreliable acquisition. By contrast, the greater vulnerability of Stars relative to Bow-Target suggests that the more demanding component was active search rather than simple target engagement. In practical terms, the patient appeared more able to orient to explicitly presented targets than to generate and sustain efficient self-initiated visuospatial exploration across the screen.

The broader clinical and neurophysiological measures addressed related but different domains [33]. CRS-R, DRS, and LCF summarized responsiveness, global disability, and staged cognitive-behavioral change, not lateralized exploratory efficiency [34]. GC was retained only as a pragmatic bedside description of communicative exchange and should not be overinterpreted as a validated psychometric outcome [35]. Similarly, P300 latency in this dataset can be read only as a non-specific marker of processing speed or higher-order cognitive efficiency [36]. Without amplitude, montage, topography, or task details, it cannot support hemispheric or spatial-attentional inferences [37].

Within these limits, the AAC/eye-tracking context still had clinical value [38]. It provided a structured communicative environment in which lateralized exploratory behavior could be repeatedly observed and partially quantified in a patient for whom conventional neglect assessment was constrained by severe impairment [39]. The data therefore support a cautious multimodal interpretation: AAC did not diagnose neglect, but it made a neglect-like exploratory pattern more observable in ecologically relevant conditions [40]. This may be especially useful when clinicians need to distinguish between failure of guided target engagement and reduced spontaneous exploration [41].

Several limitations remain decisive. This is a single-case report. The three contextual cases were descriptive only and cannot support inference. The study did not include a gold-standard neglect battery or formal visual field assessment, so coexisting hemianopic contributions could not be formally excluded. GC was not validated, and P300 characterization was incomplete. Heatmap interpretation remained qualitative, and session-to-session performance was likely modulated by vigilance, fatigue, stimulus salience, and changes in the general medical or pharmacological condition [42,43,44,45,46,47,48]. Future work should combine AAC-derived metrics with standardized neglect assessment, reproducible side-specific outputs, and larger longitudinal cohorts [49,50].

## 5. Conclusions

In this exploratory longitudinal case of hemorrhagic stroke, digital eye-gaze AAC did not provide formal diagnostic proof of neglect, but it did reveal a reproducible pattern that was clinically compatible with lateralized visuospatial attention impairment. The most defensible interpretation was preserved guided orienting with reduced spontaneous visuospatial exploration/search efficiency.

Accordingly, AAC may be useful as a structured ecological observational context in complex neurorehabilitation settings, especially when severe motor, cognitive, or communication limitations constrain conventional assessment. The present findings should be read as feasibility and potential clinical utility data rather than as validation of AAC as a stand-alone neglect measure.

## Figures and Tables

**Table 1:** Longitudinal clinical, communication, and neurophysiological findings in the index case.

Measure	T0	T1	T2	T0–T2 Change
LCF	2	3	3	+1
CRS-R	7	10	11	+4
DRS	25	25	25	0
GC	7	9	9	+2
P300 latency (ms)	393	350	351	−42 ms

Legend: LCF, Levels of Cognitive Functioning; CRS-R, Coma Recovery Scale-Revised; DRS, Disability Rating Scale; GC, Global Communication Scale. GC is reported only as a descriptive pragmatic clinical indicator.

**Table 2:** Descriptive contextual values from the additional cases assessed with the shared clinical and neurophysiological variables.

Measure	T0	T1	T2
LCF	1.67 ± 0.58	2.67 ± 0.58	2.67 ± 0.58
CRS-R	7.00 ± 3.46	9.67 ± 3.51	9.67 ± 3.51
DRS	25.33 ± 1.15	25.33 ± 1.15	25.33 ± 1.15
GC	8.00 ± 1.00	9.33 ± 1.53	9.67 ± 1.15
P300 latency (ms)	334.67 ± 28.36	329.67 ± 26.27	329.67 ± 26.27

Values are mean ± standard deviation. The contextual sample included three patients, with a mean age of 56.3 ± 9.0 years and 13 years of education in all cases. Two patients were male and one was female. These cases were used only for descriptive contextualization and did not undergo the same digital AAC/eye-tracking procedure as the index case.

**Table 3:** Serial AAC/eye-tracking process data in the index case.

Component	Observed Result	Interpretive Note
Available serial AAC/eye-tracking data	21 analyzable sessions; Blocks preliminary training completed in all sessions	Supports repeated clinical feasibility of the device in severe acquired brain injury
Calibration (coding 0 = poor, 1 = good, 2 = perfect)	Center: 19 good/2 perfect; upper-left: all good; lower-left: all good; upper-right: 8 good/13 perfect; lower-right: 19 good/2 perfect; no session scored 0	Guided orienting and target engagement were consistently at least adequate across the visual field
Stars task	Mean 2.14 hits; median 1; range 0–8; zero-hit sessions 5/21	Greater vulnerability under exploratory, self-generated search demands
Bow-Target task	Mean 3.48 hits; median 2; range 0–9; zero-hit sessions 1/21	Relatively better preserved performance in a more direct, target-bound task
Initial vs. final phase	Stars 2.70 → 1.64; Bow-Target 3.70 → 3.27	No stable longitudinal recovery of exploratory visuospatial efficiency was documented
Heatmap pattern	Non-homogeneous coverage, localized clusters, frequent rightward lateralization, reduced left-sided exploration	Consistent with reduced spontaneous visuospatial exploration under less guided conditions

Calibration coding: 0 = poor; 1 = good; 2 = perfect. Heatmap interpretation remained qualitative and was integrated with the broader clinical context because interface elements were not always uniformly distributed across screens.

## Data Availability

The data presented in this study are available on request from the corresponding author due to ethical and legal restrictions related to patient privacy and institutional data-protection requirements.

## Supplementary Materials

The following supporting information can be downloaded at: https://www.mdpi.com/article/10.3390/brainsci16050456/s1, Methods S1: Operational Definitions and Supplementary Statistical Approach for AAC/Eye-Tracking Summaries; Table S1. Raw Clinical and Neurophysiological Values for the Three Contextual Benchmark Cases; Table S2. Calibration Quality Across the Center and Four Visual Quadrants; Table S3. Additional Exact-Proportion and Variability Statistics for Stars and Bow-Target; Table S4. Representative Phase-Based Qualitative Coding of Free-Exploration Heatmaps; Figure S1. Representative Free-Exploration Heatmaps Across the Initial, Intermediate, and Final Phases.

## Abbreviations

AAC, augmentative and alternative communication; CRS-R, Coma Recovery Scale-Revised; DRS, Disability Rating Scale; GC, Global Communication Scale; LCF, Levels of Cognitive Functioning; P300, P300 event-related potential component.
