# Supplementary material for: Augmentative and Alternative Communication as an Ecological Window on Neglect-Related Spatial Asymmetry After Hemorrhagic Stroke: A Longitudinal Case Report
Source: Brain Sci. 2026 Apr 24;16(5):456. doi: 10.3390/brainsci16050456 (PMC13204121; doi:10.3390/brainsci16050456)
Supplement: Supplementary file 1 [file brainsci-16-00456-s001.zip › Supplementary Figure_S1.pdf]

Supplementary Figure S1. Representative Free-Exploration Heatmaps Across the Initial, Intermediate, and Final Phases

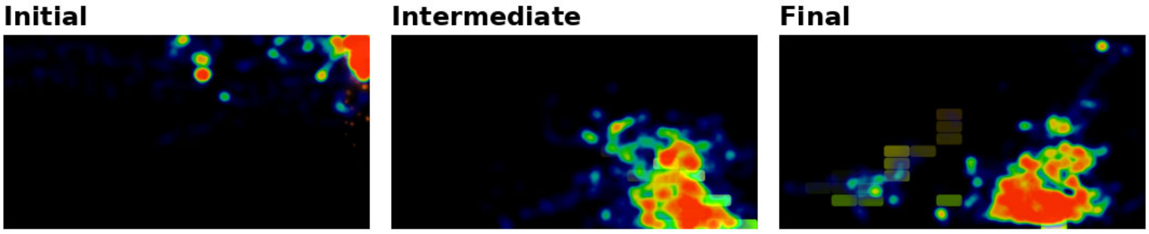

Figure legend. Representative heatmaps from the initial, intermediate, and final phases showed persistent rightward dominant clustering, reduced left-sided exploration, and limited evidence of systematic whole-screen scanning. The final-phase image showed partial leftward re-engagement, but the overall pattern remained fragmented and spatially imbalanced. These images are presented as qualitative illustrations only.
