# Supplementary material for: Augmentative and Alternative Communication as an Ecological Window on Neglect-Related Spatial Asymmetry After Hemorrhagic Stroke: A Longitudinal Case Report
Source: Brain Sci. 2026 Apr 24;16(5):456. doi: 10.3390/brainsci16050456 (PMC13204121; doi:10.3390/brainsci16050456)
Supplement: Supplementary file 1 [file brainsci-16-00456-s001.zip › Supplementary Methods S1.pdf]

Supplementary Methods S1. Operational Definitions and Supplementary Statistical Approach for AAC/Eye-Tracking Summaries

The supplementary analyses were restricted to quantities that could be derived directly from the verified source set without reconstructing or imputing unrecoverable data. Shared clinical variables for the index case and contextual benchmark cases were taken from the institutional dataset. AAC/eye-tracking summaries for the index case were taken from the verified narrative source describing 21 analyzable sessions.

Operational definitions

Calibration coding was defined as 0 = poor, 1 = good, and 2 = perfect. “At least good” referred to scores of 1 or 2. A zero-hit session was defined as a session in which the documented hit count for the task was 0. Initial-versus-final phase means were treated as phase-level descriptive summaries rather than as independent repeated observations.

Recoverable and non-recoverable task-level information

The verified source set preserved aggregate summaries for Stars and Bow-Target across 21 analyzable sessions, including means, medians, ranges, zero-hit frequencies, and initial-versus-final phase means. However, the complete paired session-by-session hit sequence for the two tasks was not recoverable from the verified source set. As a consequence, exact paired Wilcoxon comparisons, paired effect-size estimation, Theil–Sen session-level slope estimation, and Kendall rank trend analysis were not computed.

Supplementary statistical descriptors

For the recoverable task summaries, supplementary descriptors included coefficient of variation, exact binomial 95% confidence intervals for the proportion of zero-hit sessions, and absolute and relative initial-versus-final phase change. For calibration, absolute frequencies, percentages, and exact binomial 95% confidence intervals for perfect calibration were calculated for the center and each visual quadrant. These summaries were interpreted conservatively and without causal language.

Qualitative heatmap coding

Representative early, intermediate, and final free-exploration heatmaps were coded qualitatively for rightward dominant clustering, reduced left-sided exploration, non-homogeneous coverage, and the degree of whole-screen scanning. Because interface elements were not uniformly distributed and only representative images were available, this coding was treated as structured qualitative support rather than as a formal spatial metric.
