# Supplementary material for: Augmentative and Alternative Communication as an Ecological Window on Neglect-Related Spatial Asymmetry After Hemorrhagic Stroke: A Longitudinal Case Report
Source: Brain Sci. 2026 Apr 24;16(5):456. doi: 10.3390/brainsci16050456 (PMC13204121; doi:10.3390/brainsci16050456)
Supplement: Supplementary file 1 [file brainsci-16-00456-s001.zip › Supplementary Table S1.pdf]

Supplementary Table S1. Raw Clinical and Neurophysiological Values for the Three Contextual Benchmark Cases

These values are provided only for descriptive transparency. The contextual benchmark was not used as a control group and did not support inferential comparison.

Case	Age	Sex	Educ	LCF T0	LCF T1	LCF T2	CRS-R T0	CRS-R T1	CRS-R T2	DRS T0	DRS T1	DRS T2	GC T0	GC T1	GC T2	P300 T0	P300 T1	P300 T2
Case A	65	F	13	2	3	3	11	13	13	24	24	24	9	9	9	349	339	339
Case B	47	M	13	1	2	2	5	6	6	26	26	26	8	8	9	353	350	350
Case C	57	M	13	2	3	3	5	10	10	26	26	26	7	11	11	302	300	300
