# Supplementary material for: Augmentative and Alternative Communication as an Ecological Window on Neglect-Related Spatial Asymmetry After Hemorrhagic Stroke: A Longitudinal Case Report
Source: Brain Sci. 2026 Apr 24;16(5):456. doi: 10.3390/brainsci16050456 (PMC13204121; doi:10.3390/brainsci16050456)
Supplement: Supplementary file 1 [file brainsci-16-00456-s001.zip › Supplementary Table S2.pdf]

Supplementary Table S2. Calibration Quality Across the Center and Four Visual Quadrants

Calibration coding was 0 = poor, 1 = good, and 2 = perfect. No calibration score of 0 was documented in any analyzable session.

Sector	Good (n/21)	Perfect (n/21)	At least good (%)	At least good exact 95% CI	Perfect (%)	Perfect exact 95% CI
Center	19	2	100.0	83.9–100.0	9.5	1.2–30.4
Upper-left	21	0	100.0	83.9–100.0	0.0	0.0–16.1
Lower-left	21	0	100.0	83.9–100.0	0.0	0.0–16.1
Upper-right	8	13	100.0	83.9–100.0	61.9	38.4–81.9
Lower-right	19	2	100.0	83.9–100.0	9.5	1.2–30.4

The lower bound of the exact confidence interval for “at least good” reflects binomial uncertainty despite the observed 21/21 frequency.
