# Supplementary material for: Augmentative and Alternative Communication as an Ecological Window on Neglect-Related Spatial Asymmetry After Hemorrhagic Stroke: A Longitudinal Case Report
Source: Brain Sci. 2026 Apr 24;16(5):456. doi: 10.3390/brainsci16050456 (PMC13204121; doi:10.3390/brainsci16050456)
Supplement: Supplementary file 1 [file brainsci-16-00456-s001.zip › Supplementary Table S3.pdf]

Supplementary Table S3. Additional Exact-Proportion and Variability Statistics for Stars and Bow-Target

The verified source set preserved aggregated task summaries across 21 analyzable sessions. Full paired session-by-session hit sequences were not recoverable; accordingly, exact paired nonparametric tests and session-level trend estimators were not computed.

Metric	Stars	Bow-Target
Mean hits	2.14	3.48
Standard deviation	2.08	2.54
Median hits	1	2
Range	0-8	0-9
Coefficient of variation	0.97	0.73
Zero-hit sessions (n/21)	5/21	1/21
Zero-hit proportion (%)	23.8	4.8
Zero-hit exact 95% CI (%)	8.2–47.2	0.1–23.8
Initial-phase mean	2.70	3.70
Final-phase mean	1.64	3.27
Absolute change (final - initial)	-1.06	-0.43
Relative change (%)	-39.3	-11.6

These supplementary descriptors support the main-text interpretation that task performance was lower and more unstable in the more exploratory Stars condition than in the more target-bound Bow-Target condition, while remaining strictly descriptive and non-causal.
