# Supplementary material for: Augmentative and Alternative Communication as an Ecological Window on Neglect-Related Spatial Asymmetry After Hemorrhagic Stroke: A Longitudinal Case Report
Source: Brain Sci. 2026 Apr 24;16(5):456. doi: 10.3390/brainsci16050456 (PMC13204121; doi:10.3390/brainsci16050456)
Supplement: Supplementary file 1 [file brainsci-16-00456-s001.zip › Supplementary Table S4.pdf]

Supplementary Table S4. Representative Phase-Based Qualitative Coding of Free-Exploration Heatmaps

Coding was applied to the three representative images available in the verified source set (initial, intermediate, and final phases). The coding is intentionally qualitative and is not intended as a formal side-specific spatial metric.

Phase	Rightward dominant clustering	Reduced left-sided exploration	Non-homogeneous coverage	Whole-screen scanning	Brief note
Initial	Present	Present	Present	Limited	Marked clustering in the right upper field with sparse left-sided exploration.
Intermediate	Present	Present	Present	Limited	Dominant right-sided cluster persisted, mainly in the lower-right region.
Final	Present	Present but less absolute	Present	Limited	Rightward dominance persisted with partial leftward re-engagement, but coverage remained fragmented.

Because interface elements were not uniformly distributed and only representative images were recoverable, this table should be interpreted as structured qualitative support rather than as a quantitative heatmap analysis.
